# Extreme environments: crucibles of potent abiotic stress tolerance

**DOI:** 10.1093/jxb/eraa269

**Published:** 2020-06-26

**Authors:** Peter Doerner

**Affiliations:** Institute for Molecular Plant Science, School of Biological Sciences, University of Edinburgh, Edinburgh, UK

**Keywords:** Abiotic stress, commensalism and symbiosis, extreme environments, macromolecular damage, reactive oxygen species (ROS), relative growth rate, signalling

**Extreme environments, in which physical conditions for plant life can approach the limits of the biochemically possible for multicellular organisms, comprise a large fraction of the Earth’s surface. With non-linear climate change looming, bringing increasingly unpredictable weather, both the natural and the cultivated agro-ecological environment will experience increasing exposure to extreme conditions. Plants that evolved to grow in extreme environments can cope with more extreme events than generally encountered in the managed agricultural space. In this special issue, a diverse collection of reviews discusses current literature in this field, proposing some intriguing approaches inspired by plants growing in extreme environments that are relevant for cultivated plants and food security.**

Plants, as sessile organisms, cannot escape their environment; they evolved to survive by adapting to environmental and biotic adversity. Plants live in a wide variety of ecological spaces and climatic zones. Unsurprisingly, the areas of origin of the majority of domesticated and cultivated plants overlaps well with the same areas where humans have lived from Neolithic times: the human climate niche, which has remained remarkably stable over the past 6000 years (Doebley *et al.*, 2006; Xu *et al.*, 2020). The 2015 UN Climate Change Conference of 196 parties, in Paris, France, agreed on a goal of limiting global increases to 1.5 °C; but, even if these are met, they will not be uniform across the globe. Recent studies predict that by 2100, in a ‘business as usual’ scenario, up to a third of humankind will live in environments in which the mean annual temperature is 29 °C, which is currently only experienced by <1% of the Earth’s surface (Xu *et al.*, 2020). Moreover, temperature increases and the associated climate changes to the affected ecological spaces are not likely to occur gradually and linearly. Instead, change is projected to be non-linear, with acute ‘tipping points’, suggesting that change can occur quite suddenly and drastically (Trisos *et al.*, 2020). Taken together, these trends reinforce a recognition that a significant fraction of fertile soils and of global food production will be subject to the risk of increasingly extreme environmental conditions in the near future.

Much of the planet’s surface comprises ecological environments that are extreme in one or more environmental conditions. Latin America, and Chile in particular, has many such environments in which single and multicellular life including plants grow close to the limits of the biochemically possible. This insight inspired the location, theme, and the lively discussions at the meeting on ‘Integrative biology: From molecules to ecosystems in extreme environments’ held in Santiago, Chile in April 2019, which form the basis of this special issue. The meeting brought together researchers engaged in molecular, physiological, and genetic approaches to dissect and understand abiotic stress responses in many organisms, from cyanobacteria, fungi, and crop plants, to trees; and with habitats in environments from hot springs, the high-altitude desert, to subarctic forests. The articles in this special issue cover a selection of the themes and discussions at the meeting.

**Box 1. Abiotic stress and growth**

Plants evolved for fitness in diverse environments where they experience varying magnitudes of the impairment and damage to their macromolecules characteristic for abiotic stress. This has resulted in the evolution of distinct archetypical life strategies (reviewed by Lambers and Poorter, 1992). Eco-physiologists have classified plants into three main groups according to the ecological spaces they are optimally adapted to: environments with low stress with low disturbance (competitive plants), those with high stress levels, from extreme environments, but with low disturbance (stress-tolerant plants), and environments with low stress but with high disturbance levels (ruderal plants) (Grime, 1977). Domesticated and cultivated plants largely belong to the last category. Plants belonging to each of these categories share some stereotypic behaviour and traits. For example, stress-tolerant plants have low intrinsic relative growth rates, low photosynthetic rates, long-lived organs, low investment in seed production, and a low capacity for nutrient uptake. In contrast, ruderal plants (and, by extension, crops), have high intrinsic relative growth rates, are generally annuals, have high photosynthetic rates, a high investment in seed production, are good at competing, and a have high capacity for nutrient uptake (Grime, 1977; Chapin, 1991). The crucial observation is that even when provided with optimal resources, stress-tolerant plants will underperform against ruderals, particularly with respect to growth rate, resource acquisition, and photosynthetic capacity (Chapin, 1991), indicating that these growth habits have evolved and are not intra-generational adaptations.

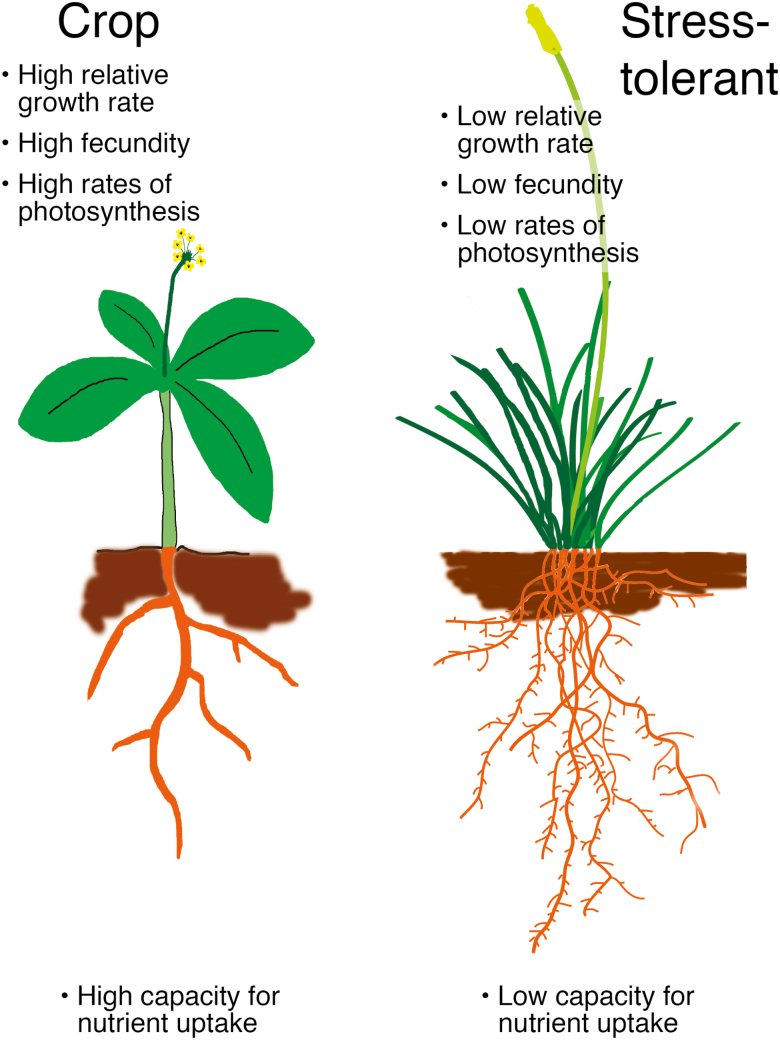

Trees are among the largest and longest living organisms on the planet; many have long generation times. In times of rapid climate change, adaptive mutations may therefore only very slowly be selected for; hence, trees are particularly at risk of damage and death by abiotic stress. Recent work has, however, shown that trees can be genetic mosaics due to the accumulation of distinct somatic mutations in the multitude of meristems in their branches, which can be transmitted (Plomion *et al.*, 2018). In this issue, Estravis-Barcala and colleagues give an extensive overview of the networks of regulatory genes and plant growth regulatory pathways involved in tree abiotic stress responses (Estravis-Barcala *et al.*, 2020). A special emphasis is given to recent advances in characterizing epigenetic responses to environmental stresses in trees and the mechanisms underlying local adaptive responses. Janni and colleagues review the responses to heat stress (Janni *et al.*, 2020). Heat shock factors (HSFs) and other transcription factors orchestrate heat shock responses, including the expression of chaperones that are important in the responses to macromolecular damage (Queitsch *et al.*, 2002; Kultz, 2005). It is intriguing that some HSFs can integrate stress responses with growth and developmental regulation; their overexpression can enhance thermotolerance and water use efficiency without a yield penalty (Bechtold *et al.*, 2013; Albihlal *et al.*, 2018). Renau-Morata and colleagues highlight recent discoveries around ‘Cycling DNA binding with One Finger’ (CDF) transcription factors that similarly reveal an integrated role in abiotic stress responses and regulation of growth and development. When overexpressed, some CDFs can provide benefits in stress and non-stress conditions, without yield penalties (Renau-Morata *et al.*, 2020). An increased capacity to mitigate degradation or damage of macromolecules, even in non-stress conditions, mediated by these and probably additional transcription factors is clearly beneficial and points towards a path to how crops with increased resilience could maintain enhanced growth performance.

The increase of the abundance of reactive oxygen species (ROS) is a common consequence of perhaps all types of abiotic stress. ROS are potent stress signalling molecules within the plant, and are also required for signalling in growth and development. One crucial metabolite required for ROS generation in signalling, but also as a cofactor for the enzymes of the anti-oxidative enzyme machinery, is NADPH, the bulk of which is synthesized in the oxidative pentose phosphate pathway (oxPPP). Chaput and co-workers describe how nitrogen (N), carbon (C), and sulfur (S) metabolism are tightly coupled through the intermediates of the oxPPP, and illuminate how a functioning oxPPP feeds back on C, N, and S assimilation and metabolism to maintain or restore homeostasis (Chaput *et al.*, 2020). However, ROS are not only signals to orchestrate plant growth, development, metabolism, and stress responses; high-level, and potentially damaging ROS accumulation, can also be the result of loss of physiological homeostasis resulting from abiotic stress. The key physiological process that is affected by abiotic stress and extreme environments in this way is photosynthesis. Oversimplified: if energy input and capture are not balanced by equivalent levels of output as chemically captured energy, the surplus energy results in rampant ROS evolution. Photosynthesis is immensely challenging for cyanobacteria and red algae, which retain the capacity to harvest light and photosynthesize in extreme environments such as hot springs with extremes of pH and temperature. Puzorjov and McCormick discuss the role of phycobilisomes, composed of phycobiliproteins, in light harvesting in extreme environments, including the blue phycobiliprotein phycocyanin which has significant biotechnological potential as a thermostable natural pigment protein (Puzorjov and McCormick, 2020).

Plant growth and development, and environmental responses, are coordinated by many plant growth regulators. Ribba and colleagues review the many roles of auxins in responses to salt stress, which both coordinate changes in growth behaviour and influence the expression of genes encoding enzymes involved in ROS detoxification (Ribba *et al.*, 2020). Recent experimental evidence highlights the importance of highly spatially regulated local auxin synthesis and precise regulation of auxin poise for growth homeostasis and tropic behaviour (Brumos *et al.*, 2018; Fendrych *et al.*, 2018).

Four reviews in this issue focus on recent advances in our understanding of how biotic interactions can modify host abiotic stress responses. While some biotic interactions are commensal or symbiotic, and help the host to respond to and cope with abiotic stress, others are detrimental and can strongly impede abiotic stress responses if the plant is already experiencing biotic stress. Silva-Sanzana and colleagues review recent progress in understanding the molecular basis of plant–aphid interactions, which are an important concern for commercial crops grown at high densities (Silva-Sanzana et al., 2019). It has become very clear over the last two decades that many multicellular organisms continuously interact with a diverse set of microorganisms, constituting a meta-organism, to the extent that many physiological functions and survival in extreme conditions would be impossible without them. These can range from the commensal fungal and bacterial interactors discussed by Pérez-Alonso and colleagues (Perez-Alonso *et al.*, 2020) and Saad and colleagues (Saad *et al.*, 2020), to the evolved symbiotic interactions between rhizobia and legumes discussed by Concha and Doerner (2020). Pérez-Alonso and colleagues highlight the exceptional properties of the endophytic, broad host range root-colonizing fungus *Serendipita indica*, which can enhance plant performance in stress conditions. Saad and colleagues review the diversity and complexity of microbial communities commensal with plants, particularly plant root systems, as endophytes or in the rhizosphere, and approaches towards assembling synthetic microbial communities for enhancing crop resilience and performance during stress. Concha and Doerner review advances in the molecular characterization of the multitude of biochemical and signalling ties that bind the rhizobium–legume symbiotic relationship.

## What can be learned from plants and algae from extreme environments to enhance resilience and performance of crops?

Plants that experience most forms of abiotic stress, singly or in combination, rapidly accumulate ROS due to loss of metabolic homeostasis resulting in the inability to optimally harness the light energy captured in photosynthesis. The resultant macromolecular damage (e.g. to proteins) can be mitigated by enhanced levels of chaperone activity and ROS-detoxifying enzymes, under control of a small number of HSFs and other transcription factors. Considering the incessant (albeit at lower intensities) exposure of plants to the physical factors that trigger abiotic stress, constitutively higher activities of master regulators orchestrating measures to counteract macromolecular damage are not detrimental in non-stress conditions. However, it remains to be determined whether, for example, xerophytes have more potent and efficient mechanisms to combat abiotic stress than plants growing in more benign environments.

The exceptional abiotic stress resilience of many plants with a stress-tolerant lifestyle is associated with their low relative growth rate. However, whether the slow growth trait is causal or just correlated to robust stress resilience is not proven. While as a global trait continuous slow growth is not desirable in high-performance crops, precise and temporally limited down-regulation of growth could prove to be a viable strategy to protect crops against extreme, but transient, adverse conditions to let them live another day and yield harvestable product. The relationship between growth control and resilience to abiotic stress merits further investigation.

Many experimental approaches to enhance abiotic stress tolerance involve the transfer of an effector gene from a heterologous stress-tolerant species, into a model or a crop plant. In only a minority of cases is the outcome a tangible enhancement of crop robust abiotic stress tolerance without a yield penalty in non-stress conditions. This can be the result of suboptimal design of the expression strategy, but also because the parachuting in of a heterologous function into an evolved physiological framework of the host plant can be ineffective or detrimental. Nonetheless, exploiting genetic diversity of known, functionally important regulators in the target species by using orthologues from adapted varieties or other, stress-tolerant, species can become a viable strategy to enhance crop performance.
